# Effect of correcting for gestational age at birth on population prevalence of early childhood undernutrition

**DOI:** 10.1186/s12982-018-0070-1

**Published:** 2018-02-06

**Authors:** Nandita Perumal, Daniel E. Roth, Johnna Perdrizet, Aluísio J. D. Barros, Iná S. Santos, Alicia Matijasevich, Diego G. Bassani

**Affiliations:** 1 Department of Epidemiology, Dalla Lana School of Public Health, University of Toronto, Toronto, ON Canada; 2 Centre for Global Child Health, Child Health Evaluative Sciences, Hospital for Sick Children, 686 Bay Street, Toronto, ON M5G 0A4 Canada; 3 Division of Paediatric Medicine, Hospital for Sick Children, Toronto, ON Canada; 4 Departments of Paediatrics and Nutritional Sciences, University of Toronto, Toronto, ON Canada; 5 Postgraduate Program in Epidemiology, Federal University of Pelotas, Pelotas, Rio Grande do Sul Brazil; 6 Department of Preventive Medicine, School of Medicine, University of São Paulo, São Paulo, Brazil

**Keywords:** World Health Organization Growth Standards (WHO-GS), Gestational age, Growth, Preterm birth, Pediatrics, INTERGROWTH newborn size standard

## Abstract

**Background:**

Postmenstrual and/or gestational age-corrected age (CA) is required to apply child growth standards to children born preterm (< 37 weeks gestational age). Yet, CA is rarely used in epidemiologic studies in low- and middle-income countries (LMICs), which may bias population estimates of childhood undernutrition. To evaluate the effect of accounting for GA in the application of growth standards, we used GA-specific standards at birth (INTERGROWTH-21st newborn size standards) in conjunction with CA for preterm-born children in the application of World Health Organization Child Growth Standards postnatally (referred to as ‘CA’ strategy) versus postnatal age for all children, to estimate mean length-for-age (LAZ) and weight-for-age (WAZ) *z* scores at 0, 3, 12, 24, and 48-months of age in the 2004 Pelotas (Brazil) Birth Cohort.

**Results:**

At birth (n = 4066), mean LAZ was higher and the prevalence of stunting (LAZ < −2) was lower using CA versus postnatal age (mean ± SD): − 0.36 ± 1.19 versus − 0.67 ± 1.32; and 8.3 versus 11.6%, respectively. Odds ratio (OR) and population attributable risk (PAR) of stunting due to preterm birth were attenuated and changed inferences using CA versus postnatal age at birth [OR, 95% confidence interval (CI): 1.32 (95% CI 0.95, 1.82) vs 14.7 (95% CI 11.7, 18.4); PAR 3.1 vs 42.9%]; differences in inferences persisted at 3-months. At 12, 24, and 48-months, preterm birth was associated with stunting, but ORs/PARs remained attenuated using CA compared to postnatal age. Findings were similar for weight-for-age *z* scores.

**Conclusions:**

Population-based epidemiologic studies in LMICs in which GA is unused or unavailable may overestimate the prevalence of early childhood undernutrition and inflate the fraction of undernutrition attributable to preterm birth.

**Electronic supplementary material:**

The online version of this article (10.1186/s12982-018-0070-1) contains supplementary material, which is available to authorized users.

## Background

Early childhood growth is an important indicator of a child’s future health and developmental potential [1, 2]. Childhood undernutrition—defined by weight and/or length/height more than 2 standard deviations below the standard population median for age and sex—has been associated with an increased risk of mortality [1, 3], poor cognitive development [4], lower school achievement [5], lower economic productivity [6], and a greater risk of adverse cardiometabolic outcomes [7–9] later in life. Undernutrition represents a major public health burden in low- and middle-income countries [10, 11]. Preterm birth, defined as births < 37 weeks gestational age (GA), has been consistently identified as a major risk factor for undernutrition in early life [12–15]. However, population-based estimates of undernutrition attributable to preterm-birth may be biased if they do not account for the GA of preterm-born children in the application of growth standards.

The World Health Organization Child Growth Standards (WHO-GS) are the most widely accepted international normative standards for evaluating postnatal growth among children less than 5 years of age [16, 17]. These standards are not directly applicable to children born preterm using postnatal age because they are based on a reference population of term-born children (births between 37 and 42 weeks GA) [18]. In clinical practice, guidelines commonly recommend correcting the postnatal age of children born preterm for the number of weeks that birth occurs prior to term gestation (40 weeks) to generate a ‘GA-corrected age’ (CA), which is then used to apply the WHO-GS up to 24 or 36 months of postnatal age [19, 20]. This strategy along with the recent publication of the GA-specific norms, INTERGROWTH-21st newborn size standards (IG-NS) [21] and INTERGROWTH-21st very preterm size at birth references (IG-VPBR) [22], provide new methods to account for GA in the standardization of anthropometric measures at birth and postnatally among children born across range of GA in population-based studies.

The importance of accounting for GA in the timescale for evaluating neonatal outcomes is well-recognized in perinatal epidemiology [23], and the implications of disregarding GA at birth in the application of growth standards/reference have been previously established in clinical settings [24, 25]. Yet, in population-based epidemiologic studies of undernutrition in LMICS, in which children born across a wide range of GA are typically included, CA is rarely used in the application of growth standards to preterm-born children [26]. Ignoring GA at birth penalizes children with shortened gestational duration and conflates those who are small but well-nourished given their GA at birth with children who have biologically meaningful deficits in nutritional status.

To quantify the implications of not accounting for GA at birth in the application of child growth standards for population-based estimates of undernutrition in LMICs, we compared the use of a ‘CA’ strategy, which applied GA-specific standards at birth in conjunction with WHO-GS using CA for preterm-born children postnatally, versus postnatal age in the application of WHO-GS for all infants (as is done conventionally), to estimate mean nutritional indices and indicators (prevalence of stunting and underweight) in a population-based cohort of term- and preterm-born children less than 5 years of age in Brazil. Additionally, we aimed to estimate the proportion of stunting and underweight attributable to preterm birth in the first 5 years of life using CA versus postnatal age strategy.

## Methods

### Study sample

Anthropometric data from the 2004 Pelotas Birth Cohort study were used. The cohort study methods have been described in detail elsewhere [27, 28]. Briefly, all births occurring in the five maternity hospitals in the urban areas of Pelotas, Brazil, from January 1st to December 31st 2004 were eligible for inclusion in the birth cohort study. Perinatal outcomes were ascertained from hospital records at the time of delivery. After birth, children were scheduled for follow-up at 3, 12, 24 and 48 months of age. Data on child anthropometry was collected at each follow-up. Follow-up rates at 3, 12, 24 and 48-month visits were high: 95.7, 93.6, 93.4, and 91.8%, respectively [27, 28]. The cohort study was approved by the Research Ethics Committee of the Medical School of the Universidade Federal de Pelotas for all follow-ups and, in addition, the World Health Organization Ethics Committee (Geneva) for data collected at birth. Ethical approval for this analysis was obtained from the Hospital for Sick Children, Toronto, and the University of Toronto, Canada.

### Measurements

#### Gestational age

GA at birth was measured by three different methods: (1) the Dubowitz score based on the physical and neurological characteristics of the newborn; (2) date of the last menstrual period (LMP), as reported on a mother’s prenatal card or self-reported during perinatal interview; and (3) ultrasonography evaluation performed before 20 weeks of pregnancy, as recorded on the mother’s prenatal card [27]. Although GA assessment using ultrasound in the first trimester is the most valid measure [29], we observed substantial selection bias due to missing data in ultrasound-based GA assessment. In addition, prenatal care facilities in Pelotas did not use standardized ultrasonography methods for GA assessment. Therefore, we created a GA variable using LMP when it was available, and Dubowtiz scores when LMP was missing or when GA assessment according to LMP was ≤ 22 or ≥ 45 weeks.

To check for plausibility of GA estimate, we used the best available normative reference/standards for GA-specific size at birth to flag implausible GA values. Using a conservative approach, observations were flagged (*n* = 215) if birthweight-for-GA was outside the range of (1) ± 2 standard deviations (SD) according to the Fenton growth curves [30] for births between 22^0/7^ and 32^6/7^ weeks and IG-NS [21] for births between 33^0/7^ and 36^6/7^, and (2) outside ± 3 SD according to the WHO-GS at age ‘0’ for children born ≥ 37 weeks. If a flagged GA was based on LMP, a plausible Dubowitz score was used (*n* = 174). To minimize the loss of data, flagged observations for which GA based on both LMP and Dubowitz was outside the ± 2 SD range (*n* = 11), were flagged again to assess if these values were within ± 3 SD of the birthweight-for-GA using either GA based on Dubowitz score (*n* = 9) or LMP (*n* = 1) since values within this range were less likely to be due data entry errors. Flagged observations were set to missing when, (1) GA estimates were outside ± 2 SD of the birthweight-for-GA range and had only one recorded GA assessment method (i.e. LMP or Dubowitz only, therefore could not be corroborated with another method of GA assessment) (*n* = 30), and (2) they were outside ± 3 SD birthweight-for-GA based on both LMP and Dubowitz (*n* = 1).

#### Age scales

Postnatal age at each follow-up visit was calculated by subtracting the date of the child’s birth from the calendar day of the visit. For children born preterm, CA in the postnatal period was calculated as the difference between the GA at birth and a full-term gestational duration of 280 days (40 weeks), and then subtracting this difference from the postnatal age: CA = [Postnatal age − (280 days − GA at birth)] [20]. For example, at 8 weeks postnatal age, a child born at 35 weeks GA would have a CA of 3 weeks and therefore would be compared to term-born children who are 3- weeks postnatal age. At birth, postmenstrual age/GA was used directly; therefore, estimates derived from using postmenstrual age at birth and CA for preterm-born children during postnatal visits are collectively referred to hereafter as ‘CA’.

#### Anthropometric measures

Methods used for anthropometric assessment of children in the Pelotas 2004 cohort were previously described [27]. Briefly, child length and weight were measured using a standardized protocol by trained interviewers who underwent standardization sessions every 3 months. Length at birth was measured with 1 mm precision using a foldable wooden length board specifically designed for the study. Birthweight was obtained from nursing records, which, in all hospitals, was measured using electronic pediatric scales with 10 g precision [27]. At 3, 12, and 24-month visits, the mother held the child for weight measurement. Maternal weight (no child) and the weight of clothes for the mother and the child were recorded separately. At the 48-month visit, the child was weighed (with minimal clothing). Weight measurements at postnatal ages were obtained using an electronic scale with 100 g precision. Maternal weight, where applicable, and weight of any remaining clothes, measured separately, were subtracted to calculate the child’s final weight.

### Application of growth standards

**Fig. 1:**
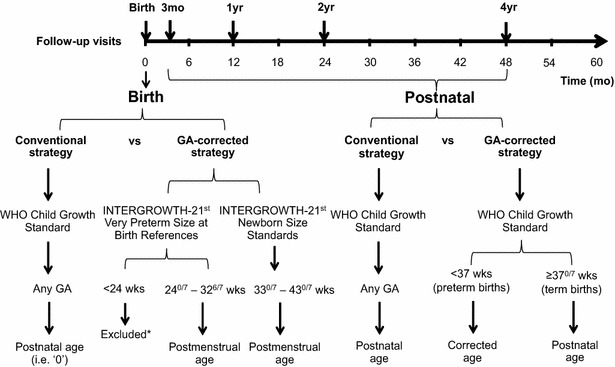
Hierarchical strategy for the application of neonatal size and postnatal growth standards using postnatal or postmenstrual timescales at various follow-up visits. * INTERGROWTH very preterm size at birth references are not applicable to infants born at < 24 week gestational age

At birth, length-for-age (LAZ) and weight-for-age (WAZ) *z* scores were derived using 0 day when applying the WHO-GS and CA when applying the IG-NS (Fig. 1). Since the IG-NS are based on a cohort of children born between 33^0/7^ and 42^6/7^ weeks GA [21], we used the IG-VPBR for children born between 24^0/7^ and < 33 weeks GA [22], and truncated births (n = 127) at > 43^0/7^ weeks GA to 300 days (43^0/7^ weeks) to permit application of the IG-NS and minimize the loss of data at birth. For one female infant born at 231 days GA, we used 232 days GA to derive *z* scores at birth because IG-NS do not estimate *z* scores for female infants born specifically at 231 days GA.

In the postnatal period beyond day 0, LAZ and WAZ were derived using the WHO-GS based on either the (1) postnatal age for all children, or (2) postnatal age for term-born children and CA for preterm-born children. In primary analyses, we did not use CA at postnatal ages for children born at early term GA (i.e., 37 or 38 weeks) or late term GA (i.e., 41 or 42 weeks) because the WHO-GS are based on a reference population of children born between 37 and 42 weeks GA and are therefore intended to be applied directly to all children born in the 5-weeks span of ‘term’ gestation. However, as a sensitivity analysis we used CA for all children (including term-born children) in the application of WHO-GS at postnatal follow-up visits.

In additional sensitivity analyses, LAZ and WAZ for preterm-born children at the 3-month visit (i.e. within 64 weeks postmenstrual age) were derived using the INTERGROWTH-21st postnatal standards for preterm-born children based on the Preterm Postnatal Follow-up Study (IG-PPFS) [31]. The WHO-GS Anthro macro (www.who.int/childgrowth/software/en/) [32] and the INTERGROWTH-21st Newborn Size Application Tool (https://intergrowth21.tghn.org/global-perinatal-package/intergrowth-21st-comparison-application/) were used for the application of the reference/standard at birth.

### Statistical analysis

Stillbirths (*n* = 56), twins (*n* = 80), or newborns with missing or implausible GA (*n* = 42) or missing both weight and length at birth (*n* = 2) were excluded from this study (see Additional file 1: Figure S1). In addition, anthropometric measurements taken outside ± 1 month of the 3-month visit (*n* = 16), outside ± 2 months of the 12- and 24-month visits (*n* = 20), and outside ± 6 months of the 48-month visit (*n* = 85) were excluded. Infants born at < 24^0/7^ weeks GA (*n* = 2) were excluded from analyses at birth as they were below the range of application of the IG-VPBR.

Repeated cross-sectional analyses were conducted to estimate and compare mean LAZ and WAZ derived using CA versus postnatal age in the application of size/growth standards at birth, 3, 12, 24 and 48-month visits. Indicators of undernutrition were the proportion of children classified as stunted (LAZ < −2 SD), underweight (WAZ < −2 SD), and wasted (weight-for-length *z* score (WLZ) < −2SD) at each visit. Paired Student’s *t* test and McNemar’s test for paired proportions were used to evaluate differences in means and proportions, respectively. The odds of undernutrition among preterm-born children (exposed) relative to term-born children (unexposed) at each follow-up visit were estimated using unadjusted logistic regression. We also determined the population attributable risk percent (% PAR) of undernutrition due to preterm birth using the following formula [33]:

$$ \left( {\frac{{Risk_{Preterm} - Risk_{Term} }}{{Risk_{Preterm} }}} \right) \times \left( {\frac{No.\; of\; preterm\; born \;children \;stunted/underweight}{Total \;no. \;of \;stunted/underweight}} \right) \times 100 $$

To further determine the effect of using CA versus postnatal age in the application of growth standards in a public health context, we simulated a population-based cross-sectional sample by randomly selecting each child at only one follow-up visit between birth and 48-months. All analyses were conducted using STATA, versions 13 and 14 Statistical Software package (StataCorp, LP).

## Results

Of 4287 births in the cohort, data from 4107 children were eligible for inclusion in this study. Children born preterm (*n* = 482; 11.7%) had a mean ± SD GA of 34.4 ± 2.5 weeks (median (interquartile range): 35 (2.0) weeks). Of the 482 children born preterm, 19.2% (*n* = 92) were born at ≤ 33^6/7^ weeks and 81.3% (*n* = 391) were born between 34^0/7^ and 36^6/7^ weeks GA. Mean ± SD GA at birth among term-born children was 39.2 ± 1.5 weeks.

As expected, children born preterm were on average shorter and lighter than term-born children at birth and at each subsequent follow-up visit up to 48-months when anthropometric indices were assessed using WHO-GS with postnatal age. However, the magnitude of the mean differences in LAZ and WAZ among term- versus preterm-born children estimated using postnatal age attenuated with increasing age (Tables 1, 2).

**Table 1:** Length-for-age *z* scores from birth to 48 months estimated using either postnatal age in the application of the World Health Organization growth child growth standards for all children, or using the INTERGROWTH 21st very preterm size at birth references, the newborn size standards at birth, and gestational age-corrected age for preterm-born children postnatally

Follow-up visit^a^	n	Excluded n		Flagged Obs^c^	Mean ± SD^d^	Median (1st, 99th)	Term-born children (≥ 37^0/7^ weeks)		Preterm-born children (< 37^0/7^ weeks)		Mean difference in LAZ among term versus preterm-born	
		No data	Ineligible for analysis				n	Mean ± SD	n	Mean ± SD	Mean difference (95% CI)	P value^e^
Birth^b^												
WHO-GS, postnatal age all	4066	41	0	23	− 0.67 ± 1.32	− 0.57 (− 5.06, 1.85)	3610	− 0.47 ± 1.07	456	− 2.25 ± 1.89	1.78 (1.61, 1.96)	< 0.001
IG-NS and IG-VPBR, CA all	4066	41	0	2	− 0.36 ± 1.19	− 0.37 (− 3.14, 2.28)	3610	− 0.37 ± 1.17	456	− 0.21 ± 1.34	− 0.17 (− 0.30, − 0.04)	0.012
3 months												
WHO-GS, postnatal age all	3859	4	2^f^	10	− 0.32 ± 1.23	− 0.24 (− 4.11, 2.24)	3444	− 0.17 ± 1.08	415	− 1.57 ± 1.61	1.41 (1.25, 1.57)	< 0.001
WHO-GS, CA for CBP, postnatal age for TBC	3859	4	2^f^	2	− 0.15 ± 1.12	− 0.11 (− 3.10, 2.34)	3444	− 0.17 ± 1.08	415	− 0.002 ± 1.34	− 0.16 (− 0.30, − 0.03)	0.016
12 months												
WHO-GS, postnatal age all	3801	3	0	3	− 0.20 ± 1.17	− 0.20 (− 3.09, 2.55)	3390	− 0.13 ± 1.15	411	− 0.76 ± 1.22	0.63 (0.50, 0.75)	< 0.001
WHO-GS, CA for CBP, postnatal age for TBC	3801	3	0	3	− 0.14 ± 1.15	− 0.14 (− 2.88, 2.56)	3390	− 0.13 ± 1.15	411	− 0.21 ± 1.19	0.08 (− 0.05, 0.20)	0.222
24 months												
WHO-GS, postnatal age all	3739	9	0	1	− 0.10 ± 1.15	− 0.09 (− 2.89, 2.56)	3324	− 0.04 ± 1.13	415	− 0.55 ± 1.20	0.50 (0.39, 0.62)	< 0.001
WHO-GS, CA for CBP, postnatal age for TBC	3739	9	0	1	− 0.07 ± 1.14	− 0.06 (− 2.86, 2.58)	3324	− 0.04 ± 1.13	415	− 0.28 ± 1.20	0.24 (0.12, 0.36)	0.001
48 months												
WHO-GS, postnatal age all	3609	4	0	1	− 0.14 ± 1.08	− 0.14 (− 2.69, 2.42)	3214	− 0.11 ± 1.07	395	− 0.42 ± 1.11	0.31 (0.19, 0.42)	< 0.001
WHO-GS, CA for CBP, postnatal age for TBC	3609	4	0	1	− 0.13 ± 1.07	− 0.13 (− 2.66, 2.43)	3214	− 0.11 ± 1.07	395	− 0.27 ± 1.11	0.17 (0.05, 0.28)	0.005

*CA* gestational age-corrected age, *CBP* children born preterm, *GA* gestational age, *IG-NS* INTERGROWTH-21st newborn size standards, *IG-VPBR* INTERGROWTH 21st very preterm size at birth references, *SD* standard deviation, *TBC* term-born children, *WHO-GS* World Health Organization child growth standards

^a^ Child postnatal age during follow-up visits ranged from: 3 months (± 1 month); 12 months (± 2 months); 24 months (± 2 months); 48 months (± 6 months)

^b^At birth,WHO-GS were applied using postnatal age to all infants born at ≥ 24^0/7^ weeks gestational age. For CA strategy, the IG-VPBR were applied to infants born between 24^0/7^ and 32^6/7^ weeks gestational age and the IG-NS were applied to infants born between 33^0/7^ and 43^0/7^ weeks gestational age. The gestational age of infants born at > 43^0/7^ was truncated 43^0/7^ (300 days) to enable application of the IG-NS

^c^Length-for-age *z* scores < −6SD or > 6SD were flagged as “biologically implausible” values by the World Health Organization child growth standards macro. These values were not excluded from summary estimates

^d^P values for difference in paired means at each visit estimated using paired sample *t* test were P < 0.001 at all follow-up visits

^e^P value for mean difference in length-for-age *z* scores among term versus preterm-born children was estimated using independent sample t-test with unequal variances

^f^The WHO-GS could not be applied to two infants who had a corrected age of < 0 at the 3-month visit and therefore were excluded from the ‘WHO-GS, postnatal age all’ strategy as well (inferences were unchanged when these two infants were included)

**Table 2:** Weight-for-age *z* scores from birth to 48 months estimated using either postnatal age in the application of the World Health Organization child growth standards for all children, or using the INTERGROWTH 21st very preterm size at birth references, the newborn size standards at birth, and gestational age-corrected age for preterm-born children postnatally

Follow-up visit^a^	n	Excluded n		Flagged Obs^c^	Mean ± SD^d^	Median (1st, 99th)	Term-born children (≥ 37^0/7^ weeks)		Preterm-born children (< 37^0/7^ weeks)		Mean difference in WAZ among term versus preterm-born	
		No data	Ineligible for analysis				n	Mean ± SD	n	Mean ± SD	Mean difference (95% CI)	P value^e^
Birth^b^												
WHO-GS, postnatal age all	4104	1	2	14	− 0.30 ± 1.20	− 0.20 (− 4.35, 1.94)	3625	− 0.09 ± 0.93	479	− 1.93 ± 1.65	1.85 (1.69, 2.0)	< 0.001
IG-NS and IG-VPBR, CA all	4104	1	2	0	0.03 ± 1.08	0.06 (− 2.48, 2.32)	3625	0.01 ± 1.07	479	0.18 ± 1.12	− 0.17 (− 0.27, − 0.06)	0.003
3 months												
WHO-GS, postnatal age all	3855	8	2^f^	6	− 0.50 ± 1.16	− 0.40 (− 3.97, 1.87)	3441	− 0.38 ± 1.04	414	− 1.56 ± 1.49	1.19 (1.04, 1.34)	< 0.001
WHO-GS, CA for CBP, postnatal age for TBC	3855	8	2^f^	0	− 0.37 ± 1.06	− 0.32 (− 3.30, 1.95)	3441	− 0.38 ± 1.04	414	− 0.29 ± 1.23	− 0.08 (− 0.21, 0.04)	0.19
12 months												
WHO-GS, postnatal age all	3779	25	0	1	− 0.25 ± 1.23	− 0.24 (− 3.27, 2.61)	3368	− 0.18 ± 1.20	411	− 0.79 ± 1.35	0.61 (0.47, 0.74)	< 0.001
WHO-GS, CA for CBP, postnatal age for TBC	3779	25	0	1	− 0.22 ± 1.22	− 0.21 (− 3.20, 2.62)	3368	− 0.18 ± 1.20	411	− 0.54 ± 1.32	0.36 (0.23, 0.50)	< 0.001
24 months												
WHO-GS, postnatal age all	3734	14	0	4	− 0.11 ± 1.21	− 0.13 (− 3.06, 2.86)	3319	− 0.06 ± 1.19	415	− 0.57 ± 1.29	0.52 (0.39, 0.65)	< 0.001
WHO-GS, CA for CBP, postnatal age for TBC	3734	14	0	4	− 0.10 ± 1.21	− 0.11 (− 2.96, 2.86)	3319	− 0.06 ± 1.19	415	− 0.41 ± 1.29	0.36 (0.22, 0.49)	< 0.001
48 months												
WHO-GS, postnatal age all	3612	1	0	11	0.43 ± 1.26	0.30 (− 2.22, 4.21)	3216	0.47 ± 1.24	396	0.05 ± 1.28	0.42 (0.29, 0.55)	< 0.001
WHO-GS, CA for CBP, postnatal age for TBC	3612	1	0	12	0.44 ± 1.25	0.31 (− 2.16, 4.23)	3216	0.47 ± 1.24	396	0.14 ± 1.29	0.33 (0.20, 0.47)	< 0.001

*CA* gestational age-corrected age, *CBP* children born preterm, *GA* gestational age, *IG-NS* INTERGROWTH-21st newborn size standards, *IG-VPBR* INTERGROWTH 21st very preterm size at birth references, *SD* standard deviation, *TBC* term-born children, *WHO-GS* World Health Organization child growth standards

^a^Child postnatal age during follow-up visits ranged from: 3 months (± 1 month); 12 months (± 2 months); 24 months (± 2 months); 48 months (± 6 months)

^b^At birth, WHO-GS were applied using postnatal age to all infants born at ≥ 24^0/7^ weeks gestational age. For CA strategy, the IG-VPBR were applied to infants born between 24^0/7^ to 32^6/7^ weeks gestational age and the IG-NS were applied to infants born between 33^0/7^ and 43^0/7^ weeks gestational age. The gestational age of infants born at > 43^0/7^ was truncated 43^0/7^ (300 days) to enable application of the IG-NS

^c^Weight-for-age *z* scores < −6SD or > 5SD were flagged as “biologically implausible” values by the World Health Organization child growth standards macro. These values were not excluded from summary estimates

^d^P values for difference in paired means estimated using a paired sample t-test at each visit were P < 0.001 at all follow-up visits

^e^P value for mean difference in weight-for-age *z* scores among term versus preterm-born children was estimated using independent sample t-test with unequal variances

^f^The WHO-GS could not be applied to two infants who had a corrected age of < 0 at the 3-month visit and therefore were excluded from the ‘WHO-GS, postnatal age all’ strategy as well (inferences were unchanged when these two infants were included)

Using IG-VPBR and IG-NS at birth (i.e., CA vs postnatal age) shifted the distributions of LAZ and WAZ higher and resulted in significantly higher mean *z* scores compared to WHO-GS. The effect was greatest among children born preterm as expected, but was also evident among term newborns (see Additional file 1: Figure S2; Tables 1, 2). Compared to estimates derived using WHO-GS at birth, distributions of LAZ and WAZ based on IG-VPBR and IG-NS had lower variance, shorter left-tails, and were more normal appearing (i.e., skewness and kurtosis values were closer to a normal distribution; data not shown). At follow-up visits, children born preterm continued to have higher mean LAZ and WAZ using CA compared to postnatal age; however, the magnitude of the effect attenuated over time (Tables 1, 2) and the distributions of LAZ and WAZ based on CA versus postnatal age were essentially overlapping by the 12 month visit (see Additional file 1: Figure S2).

At birth, using CA (vs postnatal age) attenuated the overall prevalence of stunting (8.3 vs 11.6%) and underweight (3.7 vs 6.9%) (Tables 3, 4). Similarly, at the 3-month visit, using CA versus postnatal age reduced the overall prevalence of stunting (5.1 vs 7.8%) and underweight (6.6 vs 9.3%). Although estimates varied slightly, inferences remained unchanged when CA was used for all children (including term-born children) at postnatal follow-up visits (see Additional file 1: Tables S1 and S2) or when IG-PPFS with postmenstrual age were used for preterm-born children at the 3-month visit (see Additional file 1: Tables S3 and S4). The effect of using CA for children born preterm was attenuated by the 12 month visit.

**Table 3:** Prevalence, odds of stunting (length-for-age *z* score < − 2) among children born preterm compared to term-born children, and the population attributable risk of stunting due to preterm birth, estimated using postnatal or gestational age-corrected age from birth to the 48-month follow-up visit

Follow-up visit^a^	N	Overall stunted		Term-born children (≥ 37^0/7^ weeks)		Preterm-born children (< 37^0/7^ weeks)		Odds of stunting among preterm versus term-born children	% Population attributable risk^d^
		n (%)	P^c^	n	Stunted n (%)	n	Stunted n (%)	OR (95% CI)	
Birth^b^									
WHO-GS, postnatal age all	4066	473 (11.6)	< 0.001	3610	240 (6.65)	456	233 (51.1)	14.7 (11.7, 18.4)	42.9
IG-NS and IG-VPBR, CA all	4066	337 (8.29)		3610	290 (8.03)	456	47 (10.3)	1.32 (0.95, 1.82)	3.08
3 months									
WHO-GS, postnatal age all	3859^e^	299 (7.75)	< 0.001	3444	166 (4.82)	415	133 (32.0)	9.31 (7.19, 12.1)	37.8
WHO-GS, CA for CBP, postnatal age for TBC	3859^e^	195 (5.05)		3444	166 (4.82)	415	29 (6.99)	1.48 (0.99, 2.23)	4.61
12 months									
WHO-GS, postnatal age all	3801	221 (5.81)	< 0.001	3390	160 (4.7)	411	61 (14.8)	3.52 (2.57, 4.82)	18.8
WHO-GS, CA for CBP, postnatal age for TBC	3801	191 (5.02)		3390	160 (4.7)	411	31 (7.54)	1.65 (1.11, 2.45)	6.07
24 months									
WHO-GS, postnatal age all	3739	181 (4.84)	0.002	3324	132 (4.0)	415	49 (11.8)	3.24 (2.29, 4.57)	18.0
WHO-GS, CA for CBP, postnatal age for TBC	3739	168 (4.49)		3324	132 (4.0)	415	36 (8.67)	2.30 (1.57, 3.37)	11.6
48 months									
WHO-GS, postnatal age all	3609	128 (3.55)	0.03	3214	98 (3.1)	395	30 (7.60)	2.61 (1.71, 3.99)	14.0
WHO-GS, CA for CBP, postnatal age for TBC	3609	122 (3.38)		3214	98 (3.1)	395	24 (6.08)	2.06 (1.30, 3.26)	9.80

*CA* gestational age-corrected age, *CBP* children born preterm, *CI* confidence interval, *GA* gestational age, *IG-NS* INTERGROWTH 21st newborn size standards, *IG-VPBR* INTERGROWTH 21st very preterm size at birth references, *OR* odds ratio, *PAR* population attributable risk, *TBC* term-born children, *WHO-GS* World Health Organization child growth standards

^a^ Child postnatal age during follow-up visits ranged from: 3 months (± 1 month); 12 months (± 2 months); 24 months (± 2 months); 48 months (± 6 months)

^b^ At birth, the WHO-GS were applied using postnatal age to all infants born at ≥ 24^0/7^ weeks gestational age. For CA strategy, the IG-VPBR were applied to infants born between 24^0/7^ and 32^6/7^ weeks gestational age and the IG-NS were applied to infants born between 33^0/7^ and 43^0/7^ weeks gestational age. The gestational age of infants born at > 43^0/7^ was truncated 43^0/7^ (300 days) to enable application of the IG-NS

^c^P values from McNemar’s test for difference in paired proportions when using GA-corrected strategy versus ‘WHO-GS, postnatal age all’ (reference)

^d^Proportion of all stunting in the population that is attributable to preterm

^e^The WHO-GS could not be applied to two infants who had a corrected age of < 0 at the 3-month visit and therefore were excluded from the ‘WHO-GS, postnatal age all’ strategy as well (inferences were unchanged when these two infants were included)

**Table 4:** Prevalence, odds of underweight (weight-for-age *z* score < − 2) among children born preterm compared to term-born children, and the population attributable risk of underweight due to preterm birth, estimated using postnatal or gestational age-corrected age from birth to the 48-month follow-up visit

Follow-up visit^a^	N	Overall underweight		Term-born children (≥ 37^0/7^ weeks)		Preterm-born children (< 37^0/7^ weeks)		Odds of underweight among preterm versus term-born children	% Population attributable risk^d^
		n (%)	P^c^	n	Underweight n (%)	n	Underweight n (%)	OR (95% CI)	
Birth^b^									
WHO-GS, postnatal age all	4104	284 (6.92)	< 0.001	3625	101 (2.79)	479	183 (38.2)	21.6 (16.5, 28.3)	59.7
IG-NS and IG-VPBR, CA all	4104	151 (3.68)		3625	129 (3.56)	479	22 (4.59)	1.30 (0.82, 2.07)	3.28
3 months									
WHO-GS, postnatal age all	3855^e^	359 (9.31)	< 0.001	3441	220 (6.39)	414	139 (33.6)	7.40 (5.79, 9.46)	31.3
WHO-GS, CA for CBP, postnatal age for TBC	3855^e^	254 (6.59)		3441	220 (6.39)	414	34 (8.21)	1.31 (0.90, 1.91)	2.97
12 months									
WHO-GS, postnatal age all	3779	282 (7.46)	< 0.001	3368	214 (6.35)	411	68 (16.5)	2.92 (2.18, 3.92)	14.9
WHO-GS, CA for CBP, postnatal age for TBC	3779	262 (6.93)		3368	214 (6.35)	411	48 (11.7)	1.95 (1.40, 2.71)	8.35
24 months									
WHO-GS, postnatal age all	3734	199 (5.33)	0.03	3319	157 (4.73)	415	42 (10.1)	2.27 (1.59, 3.24)	11.2
WHO-GS, CA for CBP, postnatal age for TBC	3734	193 (5.17)		3319	157 (4.73)	415	36 (8.67)	1.91 (1.31, 2.79)	8.48
48 months									
WHO-GS, postnatal age all	3612	56 (1.55)	0.24	3216	40 (1.24)	396	16 (4.04)	3.34 (1.85, 6.03)	19.8
WHO-GS, CA for CBP, postnatal age for TBC	3612	53 (1.47)		3216	40 (1.24)	396	13 (3.28)	2.70 (1.43, 5.08)	15.2

*CA* gestational age-corrected age, *CBP* children born preterm, *CI* confidence interval, *GA* gestational age, *IG-NS* INTERGROWTH 21st newborn size standards, *IG-VPBR* INTERGROWTH 21st very preterm size at birth references, *OR* odds ratio, *PAR* population attributable risk, *TBC* term-born children, *WHO-GS* World Health Organization child growth standards

^a^Child postnatal age during follow-up visits ranged from: 3 months (± 1 month); 12 months (± 2 months); 24 months (± 2 months); 48 months (± 6 months)

^b^At birth, the WHO-GS were applied using postnatal age to all infants born at ≥ 24^0/7^ weeks gestational age. For CA strategy, the IG-VPBR were applied to infants born between 24^0/7^ and 32^6/7^ weeks gestational age and the IG-NS were applied to infants born between 33^0/7^ and 43^0/7^ weeks gestational age. The gestational age of infants born at > 43^0/7^ was truncated 43^0/7^ (300 days) to enable application of the IG-NS

^c^P values from McNemar’s test for difference in paired proportions when using GA-corrected strategy versus ‘WHO-GS, postnatal age all’ (reference)

^d^Proportion of all stunting in the population that is attributable to preterm births

^e^The WHO-GS could not be applied to two infants who had a corrected age of < 0 at the 3-month visit and therefore were excluded from the ‘WHO-GS, postnatal age all’ strategy as well (inferences were unchanged when these two infants were included)

**Fig. 2:**
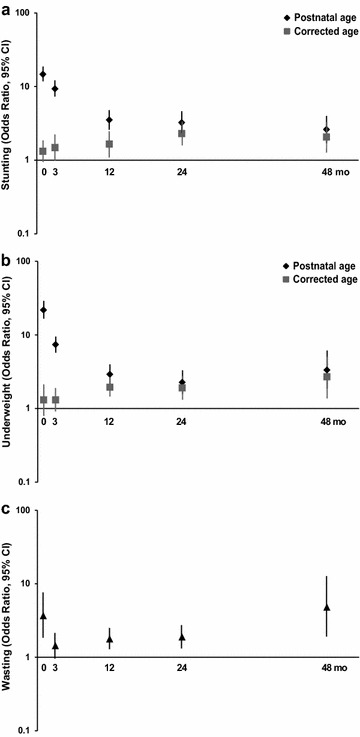
Odds ratio (95% CI) for stunting (length-for-age *z* score < −2) (**a**), underweight (weight-for-age *z* score < −2) (**b**), and wasting (weight-for-length *z* score < −2) (**c**) among preterm-born relative to term-born children < 5 years of age in the 2004 Pelotas Birth Cohort using postnatal age or corrected age in the application of WHO Child Growth Standards, in conjunction with INTERGROWTH very preterm size at birth reference and the newborn size standards. Estimates of weight-for-length *z* scores were derived using weight and length measures, independent of age. The y-axis is on the log-scale

Compared to term-born infants, children born preterm had higher estimated mean LAZ/WAZ and attenuated odds of stunting/underweight at birth and 3 months when using CA, which was opposite to the inferences based on postnatal age. From 12 to 48 months, children born preterm were at higher risks of stunting and underweight relative to term-born children when using both CA and postnatal age; however, ORs and %PARs were consistently overestimated using postnatal age (Tables 3, 4). The apparent patterns of change in ORs/%PARs with age were substantially influenced by the choice of age scale: using postnatal age, the odds ratio for stunting and underweight among preterm-born versus term-born children decreased from birth up to at least the 24-month visit, whereas using CA the odds ratios increased from birth up to at least the 24-month visit (Fig. 2). Similarly, using postnatal age, the %PAR of stunting and underweight due to preterm birth decreased from birth up to the 48-month visit, but an opposite (increasing) trend was observed when CA was used to estimate the %PAR due to preterm birth (Tables 3, 4). Of note, the magnitude of the estimates and the trends over time for stunting and underweight among children born preterm derived using CA were more closely aligned with trends in wasting, a measure of undernutrition that is estimated independently of age (Fig. 2; see Additional file 1: Table S5).

In a cross-sectional random sample of children from birth to 48 months of age, using GA-specific norms at birth alone and WHO-GS using postnatal age for all children after birth substantially attenuated the mean LAZ/WAZ and the prevalence of undernutrition at the population-level compared to using WHO-GS at birth and postnatal visits. In comparison, using CA for preterm-born children instead of postnatal age for all children when applying the WHO-GS postnatally did not greatly influence the overall estimates of the mean LAZ/WAZ or prevalence of undernutrition; however, use of postnatal age overestimated the relative odds of stunting and underweight in preterm-born versus term-born children considerably (Table 5).

**Table 5:** Effect of accounting for gestational age using gestational age-corrected age with INTERGROWTH-21st reference/standards at birth and World Health Organization growth standards postnatally, compared to using postnatal age in the application of World Health Organization growth standards at all ages on the nutritional status of children < 5 years of age in a simulated cross-sectional sample.^a^

Standard at birth	Age-scale used in the application of WHO-GS at *postnatal visits*	N	Mean ± SD	Overall undernourished^b^ n (%)	Term-born children (≥ 37^0/7^ weeks)		Preterm-born children (< 37^0/7^ weeks)		Odds of undernutrition among preterm versus term-born children	% PAR^c^
					n	Undernourished n (%)	n	Undernourished n (%)	OR (95% CI)	
Length-for-age *z* scores										
WHO-GS	Postnatal age for all	3749	− 0.30 ± 1.24	262 (6.98)	3327	158 (4.76)	422	104 (24.5)	6.54 (4.97, 8.59)	31.9
IG-VPBR and IG-NS	Postnatal age for all	3749	− 0.23 ± 1.17	231 (6.15)	3327	168 (5.05)	422	63 (14.8)	3.29 (2.41, 4.49)	17.9
IG-VPBR and IG-NS	CA for CBP, postnatal age for TBC	3749	− 0.18 ± 1.15	202 (5.39)	3327	168 (5.05)	422	34 (8.01)	1.65 (1.12, 2.42)	6.20
Weight-for-age *z* scores										
WHO-GS	Postnatal age for all	3766	− 0.18 ± 1.31	255 (6.76)	3325	150 (4.51)	441	105 (23.7)	6.62 (5.03, 8.70)	33.3
IG-VPBR and IG-NS	Postnatal age for all	3766	− 0.09 ± 1.23	213 (5.66)	3325	155 (4.65)	441	59 (13.3)	3.15 (2.29, 4.33)	17.8
IG-VPBR and IG-NS	CA for CBP, postnatal age for TBC	3766	− 0.05 ± 1.20	188 (4.99)	3325	155 (4.65)	441	33 (7.53)	1.68 (1.14, 2.47)	6.76
Weight-for-length *z* scores		3676	0.12 ± 1.24	145 (3.93)	3291	120 (3.64)	385	25 (6.46)	1.84 (1.18, 2.88)	7.55

*CA* gestational age-corrected age, *CBP* children born preterm, *CI* confidence interval, *GA* gestational age, *IG-NS* INTERGROWTH 21st newborn size standards, *IG-VPBR* INTERGROWTH 21st very preterm size at birth references, *OR* odds ratio, *PAR* population attributable risk, *TBC* term-born children, *WHO-GS* World Health Organization child growth standards

^a^Average estimates based on 500 iterations of cross-sectional samples of children randomly selected at one follow-up visit

^b^Children with length-for-age *z* scores < −2 SD (stunted) and/or weight-for-age *z* scores < −2 SD (underweight) are collectively referred to as ‘undernourished’

^c^Proportion undernourished in the population due to preterm birth

## Discussion

Using postnatal age in the application of growth standards for all children in a birth cohort in Brazil overestimated the population-average estimates of undernutrition and inflated the associations between preterm birth and undernutrition. Incorporating information about gestational duration by using CA altered indices and indicators of child nutritional status at the population-level and changed inferences with respect to the relative odds of undernutrition among children born preterm in the newborn and early infancy period. The odds of stunting and underweight due to preterm birth were diminished using CA versus postnatal age at birth; however, as expected, the effect of using CA compared to postnatal age on population-average estimates of child nutritional status progressively attenuated overtime. Beyond the 12-month follow-up, the effect of using CA on population-average cross-sectional estimates appeared to be minimal. Although the inferences at the 12- and 24-month follow-up in this study are consistent with previously reported findings from this cohort [15], the magnitude of the associations between preterm birth and stunting and underweight were attenuated when CA compared to postnatal age was used. Importantly, in contrast to estimates derived using postnatal age, we observed an increasing postnatal association between preterm birth and stunting and underweight during the first 2 years of life when CA was used. This suggests that preterm-born children in this cohort were not more likely than term infants to be undernourished at birth, but had a higher likelihood of becoming stunted and underweight beyond the early postnatal period.

As expected, using postnatal age for children born preterm in the application of WHO-GS leads to systematic bias in the measurement of nutritional status during infancy as anthropometric measures are standardized based on expected size for a given age from birth (i.e. postnatal age) as opposed to age from conception (estimated by the postmenstrual age scale). The extent to which anthropometric *z* scores are misclassified—and therefore the effect of misclassification on measures of association where child nutritional status is either the exposure or outcome—depends on the incidence of preterm birth in the source population as well as the distribution of GA among children born preterm. In LMICs where a large proportion of children are born preterm—estimated to range from ~ 10 to 18% [34]—the misclassification of infant nutritional status is non-negligible. Given the importance of gestational duration in early infancy, the effect of using CA on relative measures of associations is apparent even in the context of a cross-sectional survey in which anthropometric data are pooled from a sample of infants and young children at various ages. These findings demonstrate that population-based epidemiologic studies in LMICs [12–15] may overestimate the prevalence of undernutrition and the risk of undernutrition among preterm-born relative to term-born children, if GA is unavailable or unused in the application of growth standards.

Although the importance of using CA in analyses of anthropometric data is most apparent in early infancy (i.e., birth and 3 months), there are no known disadvantages of using CA up to 24 months of age in epidemiologic analyses, as is commonly done in clinical practice. In fact, using a GA-specific age scale in the application of growth standards confers several analytical advantages, including: (1) lower frequency of “biologically-implausible” outliers (i.e. LAZ < −6 or > 6 SD; WAZ < −6 or > 5 SD) [32], which may in part be due to incorrect assignment of *z* scores to preterm-born children, (2) reduced variance in the distribution of *z* scores leading to greater precision in the standard errors, and (3) improved normality of the *z* score distributions during early infancy.

An important limitation of this study was the use of a derived GA variable based on LMP and Dubowitz methods. These methods for GA assessment are less precise in comparison to first or second trimester ultrasound assessment. To minimize the risk of measurement error, we excluded all observations with implausible birthweight and GA combinations, which may have resulted in some selection bias by excluding observations at extreme ends of the birthweight-for-GA distribution. However, given that available ultrasound measurements in this cohort were prone to selection bias for preterm births, the systematically derived GA estimate provided a more reliable measure of GA for this study. Importantly, inferences regarding the effect of using CA compared to postnatal age for preterm-born children in the application of growth standards are robust against GA estimation methods. The effect of using CA for children born preterm in the application of WHO-GS in this study was likely a conservative estimate given the lower prevalence of undernutrition in Pelotas, Brazil, compared to countries in Sub-Saharan Africa and South Asia [10]. In addition, due to the repeated serial cross-sectional design of our analysis, we did not analyze individual-level longitudinal patterns of growth among preterm-born children using different age-scales in the application of growth standards. Furthermore, although we used the best available standards at each follow-up visit (e.g., IG-NS, IG-PPFS, and WHO-GS), it was difficult to fully disentangle the implications of using different standards (INTERGROWTH-21st vs WHO-GS) from GA-correction effect alone for comparisons at birth. However, the effects of using CA for preterm-born children (or using CA for *all* children in sensitivity analyses) at the 3-month and later visits were assessed using only WHO-GS, and therefore demonstrated that observed differences in CA versus postnatal age estimates were not an artifact of differences in standards but a true effect of accounting for GA in the standardization of anthropometric indices and indicators. Nonetheless, further research is needed to fully explore the implications of combining multiple different standards for longitudinal assessment of child growth trajectories.

In summary, this study suggests that the choice of age scale used to standardize anthropometric measures has substantial and meaningful implications for population-level epidemiologic inferences related to patterns of growth faltering and risk factors that contribute to undernutrition in early life. In LMICs with concurrently high prevalence of undernutrition and high incidence of preterm births, the discrepancies between postnatal age versus CA estimates of undernutrition at the population-level would be expected to be greater than the effect observed in this study, and may potentially misguide public health intervention priorities to reduce the population burden of undernutrition Future epidemiologic studies should therefore collect high-quality information about GA whenever possible and incorporate the GA information in the analyses of anthropometric data, at least at birth and during infancy. Longitudinal studies that account for heterogeneity in GA at birth in the application of growth standards are needed to better understand postnatal growth trajectories among children born preterm, the contribution of preterm birth to undernutrition in early life, and the implications for health outcomes in later life.

## Additional file

**Additional file 1.** Supplementary figures and tables.

## Abbreviations

CA: gestational-age corrected age
GA: gestational age
INTERGROWTH-21st: International Fetal and Newborn Growth Consortium for the 21st century
IG-NS: INTERGROWTH-21st newborn size standards
IG-PPFS: INTERGROWTH-21st postnatal standards for preterm born children
IG-VPBR: INTERGROWTH-21st very preterm size at birth references
LAZ: length-for-age *z* scores
LMP: last menstrual period
WAZ: weight-for-age *z* scores
PAR: population attributable risk
WFLZ: weight-for-length *z* score
WHO-GS: World Health Organization Growth Standards
