# Supplementary material for: Effect of correcting for gestational age at birth on population prevalence of early childhood undernutrition
Source: Emerg Themes Epidemiol. 2018 Feb 6;15:3. doi: 10.1186/s12982-018-0070-1 (PMC5799899; doi:10.1186/s12982-018-0070-1)
Supplement: Supplementary file 1 — Additional file 1. Supplementary figures and tables. [file 12982_2018_70_MOESM1_ESM.pdf]

Effect of correcting for gestational age at birth on population prevalence of early childhood undernutrition

Nandita Perumal, Daniel E. Roth, Johnna Perdrizet, Aluísio J D Barros, Iná S Santos, Alicia Matijasevich, Diego G Bassani

ADDITIONAL SUPPORTING MATERIAL

Emerging Themes in Epidemiology

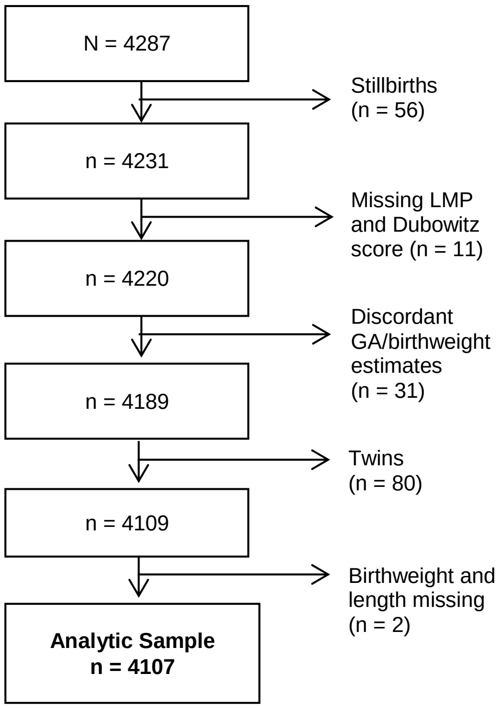

Figure S1. Flowchart of analytical sample for this study.

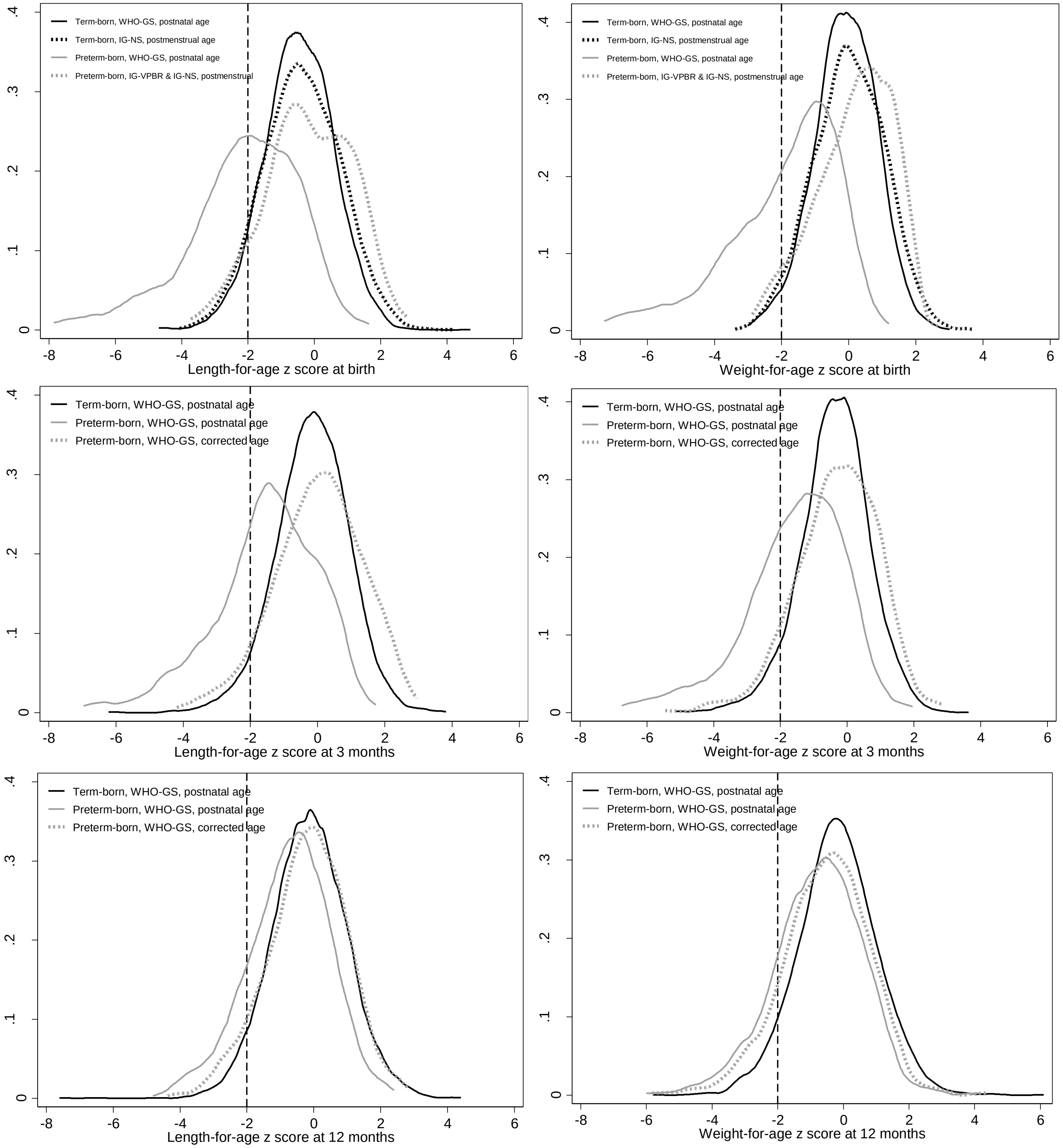

Figure S2. Comparison of the distribution of length-for-age and weight-for-age *z* scores at birth (top row), 3 months (middle row), and 12 months (bottom row) among preterm- and term-born children using postnatal age and gestational age-corrected age in the application of World Health Organization child growth standards (WHO-GS). At birth, the INTERGROWTH newborn size standards (IG-NS) and the INTERGROWTH very preterm born size at birth references (IG-VPBR) were applied for all children using postmenstrual age.

Table S1. Length-for-age *z* scores at postnatal follow-up visits estimated using either postnatal age or gestational age-corrected age in the application of the World Health Organization growth child growth standards for all children.

Follow-up visit[a]	n	Excluded n		Flagged Obs[b]	Mean ± SD[c]	Median (1st, 99th)	Term-born children (≥37 0/7 wk)		Preterm-born children (<37 0/7 wk)		Mean difference in LAZ among term vs preterm-born	
		No data	Ineligible for analysis				n	Mean ± SD	n	Mean ± SD	Mean difference (95% CI)	*P* value[d]
**3 mo[e]**												
WHO-GS, postnatal age all	3859	4	2	10	-0.32 ± 1.23	-0.24 (-4.11, 2.24)	3444	-0.17 ± 1.08	415	-1.57 ± 1.61	1.41 (1.25, 1.57)	<0.001
WHO-GS, CA all	3859	4	2	0	-0.03 ± 1.14	0.01 (-3.05, 2.46)	3444	-0.04 ± 1.12	415	-0.002 ± 1.34	-0.03 (-0.17, 0.10)	0.618
**12 mo**												
WHO-GS, postnatal age all	3801	3	0	3	-0.20 ± 1.17	-0.20 (-3.09, 2.55)	3390	-0.13 ± 1.15	411	-0.76 ± 1.22	0.63 (0.50, 0.75)	<0.001
WHO-GS, CA all	3801	3	0	3	-0.10 ± 1.16	-0.10 (-2.84, 2.58)	3390	-0.09 ± 1.16	411	-0.21 ± 1.19	0.12 (0.001, 0.25)	0.048
**24 mo**												
WHO-GS, postnatal age all	3739	9	0	1	-0.10 ± 1.15	-0.09 (-2.89, 2.56)	3324	-0.04 ± 1.13	415	-0.55 ± 1.20	0.50 (0.39, 0.62)	<0.001
WHO-GS, CA all	3739	9	0	1	-0.03 ± 1.15	-0.02 (-2.82, 2.64)	3324	-0.004 ± 1.14	415	-0.28 ± 1.20	0.28 (0.16, 0.41)	<0.001
**48 mo**												
WHO-GS, postnatal age all	3609	4	0	1	-0.14 ± 1.08	-0.14 (-2.69, 2.42)	3214	-0.11 ± 1.07	395	-0.42 ± 1.11	0.31 (0.19, 0.42)	<0.001
WHO-GS, CA all	3609	4	0	1	-0.11 ± 1.08	-0.13 (-2.63, 2.43)	3214	-0.09 ± 1.07	395	-0.27 ± 1.11	0.18 (0.06, 0.29)	0.003

CA, gestational age-corrected age; CBP, children born preterm; GA, gestational age; IG-NS, INTERGROWTH-21st newborn size standards; IG-VPBR, INTERGROWTH 21st very preterm size at birth references; SD, standard deviation; TBC, term-born children; WHO-GS, World Health Organization child growth standards

[a] Child postnatal age during follow-up visits ranged from: 3 months (± 1 month); 12 months (± 2 months); 24 months (±2 months); 48 months (± 6 months)

[b] Length-for-age *z* scores <-6SD or >6SD were flagged as "biologically implausible" values by the World Health Organization child growth standards macro. These values were not excluded from summary estimates.

[c] P-values for difference in paired means at each visit estimated using paired sample t-test were p<0.001 at all follow-up visits.

[d] P-value for mean difference in length-for-age *z* scores among term versus preterm-born children was estimated using independent sample t-test with unequal variances.

[e] The WHO-GS could not be applied to two infants who had a corrected age of <0 at the 3-month visit and therefore were excluded from the 'WHO-GS, postnatal age all' strategy as well (inferences were unchanged when these two infants were included).

Table S2. Weight-for-age *z* scores at postnatal follow-up visits estimated using either postnatal age or gestational age-corrected age in the application of the World Health Organization growth child growth standards for all children.

Follow-up visit[a]	n	Excluded n		Flagged Obs[b]	Mean ± SD[c]	Median (1st, 99th)	Term-born children (≥37$^{0/7}$ wk)		Preterm-born children (<37$^{0/7}$ wk)		Mean difference in WAZ among term vs preterm-born	
		No data	Ineligible for analysis				n	Mean ± SD	n	Mean ± SD	Mean difference (95% CI)	*P* value[d]
**3 mo[e]**												
WHO-GS, postnatal age all	3855	8	2[f]	6	-0.50 ± 1.16	-0.40 (-3.97, 1.87)	3441	-0.38 ± 1.04	414	-1.56 ± 1.49	1.19 (1.04, 1.34)	<0.001
WHO-GS, CA all	3855	8	2[f]	0	-0.28 ± 1.08	0.24 (-3.23, 2.11)	3441	-0.28 ± 1.06	414	-0.29 ± 1.23	0.02 (-0.09, 0.13)	0.763
**12 mo**												
WHO-GS, postnatal age all	3779	25	0	1	-0.25 ± 1.23	-0.24 (-3.27, 2.61)	3368	-0.18 ± 1.20	411	-0.79 ± 1.35	0.61 (0.47, 0.74)	<0.001
WHO-GS, CA all	3779	25	0	1	-0.20 ± 1.22	-0.19 (-3.15, 2.72)	3368	-0.16 ± 1.21	411	-0.54 ± 1.32	0.38 (0.26, 0.51)	<0.001
**24 mo**												
WHO-GS, postnatal age all	3734	14	0	4	-0.11 ± 1.21	-0.13 (-3.06, 2.86)	3319	-0.06 ± 1.19	415	-0.57 ± 1.29	0.52 (0.39, 0.65)	<0.001
WHO-GS, CA all	3734	14	0	4	-0.08 ± 1.21	-0.10 (-2.98, 2.85)	3319	-0.04 ± 1.19	415	-0.41 ± 1.29	0.37 (0.25, 0.49)	<0.001
**48 mo**												
WHO-GS, postnatal age all	3612	1	0	11	0.43 ± 1.26	0.30 (-2.22, 4.21)	3216	0.47 ± 1.24	396	0.05 ± 1.28	0.42 (0.29, 0.55)	<0.001
WHO-GS, CA all	3612	1	0	13	0.44 ± 1.26	0.32 (-2.20, 4.25)	3216	0.48 ± 1.25	396	0.14 ± 1.29	0.34 (0.21, 0.47)	<0.001

CA, gestational age-corrected age; CBP, children born preterm; GA, gestational age; IG-NS, INTERGROWTH-21st newborn size standards; IG-VPBR, INTERGROWTH 21st very preterm size at birth references; SD, standard deviation; TBC, term-born children; WHO-GS, World Health Organization child growth standards

[a] Child postnatal age during follow-up visits ranged from: 3 months (± 1 month); 12 months (± 2 months); 24 months (±2 months); 48 months (± 6 months)

[b] Weight-for-age *z* scores <-6SD or >6SD were flagged as "biologically implausible" values by the World Health Organization child growth standards macro. These values were not excluded from summary estimates.

[c] P-values for difference in paired means at each visit estimated using paired sample t-test were p<0.001 at all follow-up visits.

[d] P-value for mean difference in length-for-age *z* scores among term versus preterm-born children was estimated using independent sample t-test with unequal variances.

[e] The WHO-GS could not be applied to two infants who had a corrected age of <0 at the 3-month visit and therefore were excluded from the 'WHO-GS, postnatal age all' strategy as well (inferences were unchanged when these two infants were included).

Table S3. Length/weight-for-age *z* scores at the 3-month visit estimated using postnatal age in the application of the World Health Organization child growth standards for all children (WHO-GS), or accounting for gestational age using the INTERGROWTH 21st postnatal standards for preterm born children in conjunction with WHO-GS for term-born children.

Anthropometric indices at 3 months	n	Mean (SD)[a]	Median (1st, 99th)	Term-born children ($\geq 37^{0/7}$ wks)		Preterm-born children ($<37^{0/7}$ wks)		*P* value[b]
				n	Mean ± SD	n	Mean ± SD	
**Length-for-age z-score**								
WHO-GS, postnatal age all	3861	-0.32 (1.24)	-0.24 (-4.11, 2.24)	3444	-0.17 (1.08)	417	-1.59 (1.63)	<0.001
IG-PPFS, postmenstrual age for CBP; WHO-GS postnatal age for TBC	3861	-0.11 (1.14)	-0.09 (-3.10, 2.45)	3444	-0.17 (1.08)	417	0.34 (1.43)	<0.001
**Weight-for-age z-score**								
WHO-GS, postnatal age all	3857	-0.51 (1.16)	-0.40 (-4.01, 1.87)	3441	-0.38 (1.04)	416	-1.58 (1.50)	<0.001
IG-PPFS, postmenstrual age for CBP; WHO-GS postnatal age for TBC	3857	-0.32 (1.08)	-0.27 (-3.29, 2.05)	3441	-0.38 (1.04)	416	0.16 (1.26)	<0.001

[a] P-value for difference in paired means (ref: WHO-GS, postnatal age all) estimated using paired t-test were p<0.001.
[b] P-value for mean difference among term versus preterm-born children using independent sample t-test with unequal variances.
CBP, children born preterm; IG-PPFS, INTERGROWTH 21st postnatal standards for preterm born children; SD, standard deviation; TBC, term-born children; WHO-GS, World Health Organization Child Growth Standards

Table S4. Prevalence, odds of stunting (length-for-age *z* score <-2) and underweight (weight-for-age *z* scores <-2) among preterm compared to term-born children, and the population attributable risk of stunting due to preterm birth, estimated using postnatal or postmenstrual age at 3 months in the 2004 Pelotas Birth Cohort.

**Anthropometric indices at 3 months**	**n**	**Overall undernutrished[a]**		**Term-born children (≥37$^{0/7}$ wks)**		**Preterm-born children (<37$^{0/7}$ wks)**		**Odds of undernutrition among preterm vs term-born children**	**% PAR[c]**
		**n (%)**	**P value[b]**	**n**	**Undernourished n (%)**	**n**	**Undernourished n (%)**	**OR (95% CI)**	
**Length-for-age z-score**									
WHO-GS, postnatal age all	3861	301 (7.80)	<0.001	3444	166 (4.82)	417	135 (32.4)	9.45 (7.31, 12.23)	38.2
IG-PPFS, postmenstrual age for CBP; WHO-GS postnatal age for TBC	3861	190 (4.92)		3444	166 (4.82)	417	24 (5.76)	1.21 (0.78, 1.87)	2.05
**Weight-for-age z-score**									
WHO-GS, postnatal age all	3857	361 (9.36)	<0.001	3441	220 (6.39)	416	141 (33.9)	7.51 (5.89, 9.59)	31.7
IG-PPFS, postmenstrual age for CBP; WHO-GS postnatal age for TBC	3857	240 (6.22)		3441	220 (6.39)	416	20 (4.81)	0.74 (0.47, 1.18)	-2.75

[a] Children with length-for-age z-scores <-2 SD (stunted) and/or weight-for-age z-scores <-2 SD (underweight) are collectively referred to as 'undernourished'.
[b] P-values from McNemar's test for difference in paired proportions (ref: WHO-GS, postnatal age all) were p<0.001.
[c] Proportion undernourished in the population due to preterm birth.
CA, gestational age-corrected age; CBP, children born preterm; CI, confidence interval; GA, gestational age; IG-NS, INTERGROWTH 21st newborn size standards; IG-PPFS, INTERGROWTH 21st postnatal standards for preterm born children; OR, odds ratio; PAR, population attributable risk; TBC, term-born children; WHO-GS, World Health Organization child growth standards

Table S5. Prevalence, unadjusted odds of wasting (weight-for-length z score <-2) among children born preterm compared to term-born children using the World Health Organization child growth standards, and the population attributable risk of wasting due to preterm birth from birth to the 48-month follow-up visit in the 2004 Pelotas Birth Cohort.

**Follow-up visit**[a]	**n**	**Overall wasted n (%)**	**Term-born children ($\geq 37^{0/7}$ wks)**		**Preterm-born children ($<37^{0/7}$ wks)**		**Odds of undernutrition among preterm vs term-born children**		**% PAR**[b]
			**n**	**Wasted n (%)**	**n**	**Wasted n (%)**	**OR (95% CI)**	***P* value**	
**Birth**	3764	44 (1.17)	3479	34 (0.98)	285	10 (3.51)	3.68 (1.80, 7.54)	<0.001	16.4
**3 mo**	3856	193 (5.01)	3441	165 (4.80)	415	28 (6.75)	1.44 (0.95, 2.17)	0.087	4.20
**12 mo**	3777	275 (7.28)	3366	228 (6.77)	411	47 (11.4)	1.78 (1.27, 2.48)	0.001	6.97
**24 mo**	3726	195 (5.23)	3312	159 (4.80)	414	36 (8.70)	1.89 (1.30, 2.75)	0.001	8.27
**48 mo**	3606	19 (0.53)	3211	12 (0.37)	395	7 (1.77)	4.81 (1.88, 12.29)	0.001	29.1

[a] Child postnatal age during follow-up visits ranged from: 3 months (± 1 month); 12 months (± 2 months); 24 months (±2 months); 48 months (± 6 months)
[b] Proportion of all wasting in the population that is attributable to preterm births.
CI, confidence interval; OR, odds ratio; PAR, population attributable risk.
